# The Single‐Delayed‐Phase Contrast Computed Tomography Before Ablation to Reduce Radiation Exposure Without Compromising Diagnostic Pulmonary Vein Accuracy

**DOI:** 10.1111/jce.70002

**Published:** 2025-07-18

**Authors:** Keishiro Yagyu, Yasushi Oginosawa, Takahiro Kobayashi, Yuki Nakamura, Nozomu Ishii, Taro Miyamoto, Katsuhide Hayashi, Hisaharu Ohe, Masaharu Kataoka

**Affiliations:** ^1^ The Second Department of Internal Medicine University of Occupational and Environmental Health Kitakyushu Fukuoka Japan; ^2^ Department of Heart Rhythm Management University of Occupational and Environmental Health Kitakyushu Japan

**Keywords:** atrial fibrillation, catheter ablation, computed tomography, pulmonary vein

## Abstract

**Introduction:**

Early‐phase contrast‐enhanced CT (CCT) is often used to plan and guide catheter ablation for atrial fibrillation (AF), and delayed‐phase images can be used to detect or exclude left atrial appendage (LAA) thrombosis. However, dual‐phase CCT is associated with concerns about radiation exposure; hence, this study aimed to evaluate whether single‐delayed‐phase images can provide sufficient preoperative information while minimizing radiation exposure.

**Methods and Results:**

A total of 102 patients who underwent dual‐phase CCT were analyzed for pulmonary vein (PV) anatomy and LAA thrombus detection. The decrease in image quality due to the difference between early and delayed phases in 3D reconstruction did not pose a problem regarding anatomical evaluation. The PV anatomy was classified as typical or atypical, and 399 PVs were analyzed. Atypical PVs included 17 cases, with consistent anatomical details across the early and delayed phases. The mean discrepancy in PV diameter between the two phases and the correlation coefficient for the coronal view was 0.78 ± 0.16 mm, *r* = 0.91, and for the axial view, 0.79 ± 0.15 mm, *r* = 0.93. The LAA thrombi were observed in three patients, and no thrombus was overestimated in the delayed phase. The total exposure dose was 2320.1 ± 1031.0 mGy‐cm in the dual‐phase, 1443.3 ± 578.5 mGy‐cm in the single early phase, and 876.8 ± 526.6 mGy‐cm in the single delayed phase. Radiation doses were significantly lower in single‐phase imaging than in dual‐phase.

**Conclusions:**

The single‐delayed‐phase CCT provides accurate anatomical and thrombus evaluations while significantly reducing radiation exposure. This approach could be a safer alternative for pre‐ablation assessment without compromising diagnostic reliability. **Trial Registration:** The University of Occupational and Environmental Health ethics committee approved the study (UOEHCRB22‐067).

## 1 Introduction

Atrial fibrillation (AF) is one of the most prevalent arrhythmias. It is associated with an elevated risk of cardiovascular mortality and morbidity predominantly caused by atrial thrombi and irregular tachycardia [1, 2]. The electrical isolation of the pulmonary vein (PV) with transcatheter ablation is an increasingly used treatment for AF, with a success rate of maintaining sinus rhythm ranging from 62% to 90% and reducing cardiovascular morbidity [3]. An accurate assessment of the anatomy and variations in the left atrium (LA) and PVs is essential for successful ablation procedures.

Before undergoing AF ablation, contrast‐enhanced computed tomography (CCT) is performed to obtain essential information for the success of the procedure, as PV variants are common [4]. Measurement of the PV diameter and assessment of its morphology via CCT are crucial because they enable preoperative approaches tailored to individual anatomical variations. Today, the energy sources used for ablation have diversified, as have the types and shapes of devices, so it is believed that the evaluation of anatomical morphology will continue to be important in selecting the optimal treatment plan. Furthermore, it facilitates enhanced procedural safety, reduces the risk of complications, such as PV stenosis, and allows assessment of the risk of AF recurrence. Recently, CCT has been employed as a novel means of assessment [5, 6]. Dual‐phase CCT was used to evaluate the left atrial appendage (LAA), a small pouch‐like structure within the LA, and the presence of a thrombus. A thrombus in the LAA is a potential complication of AF and a significant risk factor for embolic events, such as stroke. Therefore, LAA thrombi must be excluded with the utmost reliability before ablation; delayed‐phase CCT is an invaluable tool for this assessment.

Conversely, CCT has several potential issues, the most significant of which is the increased radiation dose. CT scans involve exposure to ionizing radiation, which carries a small but cumulative risk of harm, including an increased risk of cancer [7]. Despite efforts to minimize radiation exposure, this is unavoidable to some extent as long as imaging is performed, and it remains a concern. Therefore, sufficient information must be obtained from the smallest possible number of scans.

This study aimed to determine whether it is feasible to reduce the number of CT scans to a single examination, which is typically conducted twice, using a standard protocol. To achieve this, an anatomical assessment was performed using delayed‐phase CCT compared with early‐phase CCT.

## 2 Methods

### 2.1 Patient Selection

This single‐center study investigated 102 consecutive patients who underwent ablation at the Department between June 2021 and August 2022 at the University of Occupational and Environmental Health (UOEH). This study was approved by the UOEH Ethics Committee (UOEHCRB22‐067). As this was a retrospective study, the Institutional Review Board waived the requirement for written informed consent.

### 2.2 Study Protocol

To compare the values obtained from the delayed‐phase CCT with those obtained from the early‐phase CCT, the values in the coronal and sagittal planes of each PV at each phase were measured. The two physicians who performed the measurements were trained to measure each parameter in 30 cases. Independent physicians mindlessly performed the measurements; if the difference in values exceeded the minimum one standard deviation (SD) value, a third physician was asked to perform the measurements again, and the values were determined after consultation with the physicians. Data on age, sex, left atrial diameter, duration of AF, cardiac rhythm during CT scanning, and CHADS2 score were empirically collected as predictors of PV diameter. Transesophageal echocardiography (TEE) was performed to rule out LAA thrombus in patients suspected of having a thrombus.

### 2.3 Pre‐Procedure Dual‐Phase Computed Tomography

For each patient, CT was performed using a dual‐phase multidetector CT scanner (Aquilion PRIME, CANNON, Japan) equipped with 80‐detector row units.

The nonionic contrast agent was injected using the dilution test injection method. The split dose was adjusted to the patient's weight to 20 mg/kg. During the test injection, the peak of the time‐enhanced curve was obtained by monitoring the LA. The amount of contrast agent and injection rate were determined based on the peak CT value and time obtained during the test injection. In the initial phase, the CT scan was initiated when the CT value of the LA was 300 HU and covered the entire R−R interval phase during expiration, from the apex of the heart to the lung fields. The image was acquired with a 320 mm field of view and later expanded to 160−240 mm. The parameters were: tube voltage of 120 kV, patient‐specific tube current controlled by CT‐Auto Exposure Control (CT‐AEC), image quality setting of SD 19/0.5 mm, helical pitch automatically adjusted to the cardiac rhythm, slice width of 0.5 mm, table speed variable according to the helical pitch, and tube rotation speed of 0.35 s/rotation. The scan was performed while breath‐holding. In the delayed‐phase, imaging was started 1 min after contrast injection. The imaging process was similar to the first one; however, images were captured from the apex to the roof of the heart, and the CT‐AEC image quality was set to SD 23.

Prospective electrocardiographic gating was performed to eliminate cardiac motion artifacts, and images were constructed in the left atrial dilatation layer. Image reconstruction was performed using the FC‐43 function and AIDR STR. Data reconstruction was performed on a postprocessing workstation (ZIOSTATION 2, AMIN, Japan) using two‐dimensional viewing modes and three‐dimensional (3D) reconstruction.

### 2.4 Assessment of PV Anatomy, PV Diameter, and LAA Thrombus

**Figure 1:**
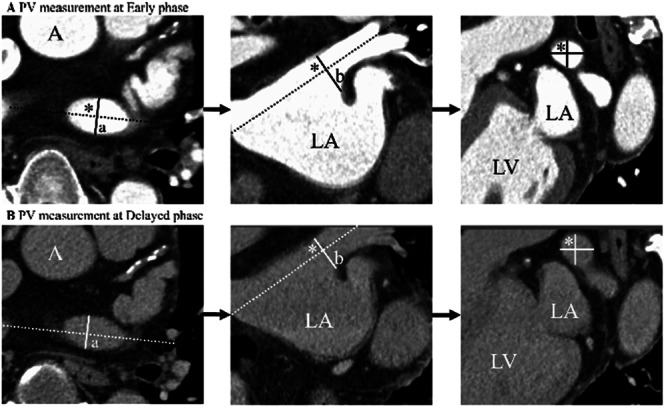
Shows the measurement of PV diameter (A) Early phase and (B) Delayed phase. Axial image and coronal image show that the crosshair on the left superior pulmonary vein at the ostium. Oblique coronal image shows the left superior pulmonary vein in cross section perpendicular to the long axis of the vessel. From this view, both the short‐axis diameter and the cross‐sectional area can be measured. Diameter and the cross‐sectional area can be measured. A, ascending aorta; LA, left atrium; LAA, Left atrium appendage; LV, left ventricle. a, PV diameter of axial axis; b, PV diameter of coronal axis. Cross‐sectional area (CSA) = π*a*b, venous ostium index (VOI) = a/b.

The PV anatomy was evaluated in three orthogonal planes (transverse, sagittal, and coronal) with angulated multiplanar reformatting as previously reported [8, 9]. In addition, we used CARTO 3 (Biosense Webster, Diamond Bar, CA, USA) to upload CCT images of the early and delayed phases, and performed anatomical evaluation. The number of PVs, two diameter measurements of each PV, the shape of each PV ostium, and PV drainage patterns were assessed (Figure 1). The early and delayed phases were evaluated at least 1 day apart to prevent bias. The shape of the PV ostium was defined using the venous ostium index and calculated as the ratio between the coronal and axial axes of each PV. The cross‐sectional area of each PV was calculated as π*(coronal axis/2)*(axial axis/2). The PV drainage pattern was classified as early PV branching, separate or common insertion of the ipsilateral PVs into the LA, or the presence of additional PVs. If a PV branch was measured within 5 mm of its insertion into the LA, it was classified as early branching [9]. Furthermore, to evaluate the positional accuracy of inferior PVs, the eighth thoracic vertebra (Th8) was used as a reference point. In cases with normal anatomy, 3D distances were measured between the bottom of the inferior PVs and the anterosuperior point of Th8. Additionally, we compared these measurements between the early and delayed phases. The presence of LAA thrombus was also independently evaluated in both phases. At least two cardiologists performed the measurements to ensure data accuracy, and the mean of the measurements was used.

### 2.5 Statistical Analysis

All calculations were performed using JMP Pro Statistical Discovery Software version 16.0. (SAS Institute Inc., Cary, NC, USA). All categorical variables are presented as proportions and continuous variables as mean ± SD. Numerical variables are presented as means and SD and rendered as mean ± SD of the difference in measurements between the delayed‐phase CCT and early‐phase CCT. Paired and Student's *t*‐tests were used to compare continuous variables, as appropriate. Fisher's exact test was used to compare categorical variables. The Kappa statistic was used to assess the agreement between the early and delayed phases in detecting PV anatomical variation. Finally, Bland–Altman plotted the PV diameter differences over the averaged values of the early‐ and delayed‐phase measurements for each individual. To assess the agreement between early‐ and delayed‐phase measurements of PV diameters, the intra‐class coefficient correlation (ICC) were computed. The significance level was set at 0.05 for all tests.

## 3 Results

### 3.1 Patient Characteristics

Table 1 presents the characteristics of the participants. All 102 patients aged 68.7 ± 9.5 years underwent CCT before PV ablation. Among them, 66 (64.7%) were men, 43 (42.2%) had paroxysmal AF, 30 (29.4%) had persistent AF, and 29 (28.4%) had other arrhythmias. The mean CHADS2 score was 1.3 points. The mean LA diameter was 39.5 ± 7.3 mm in the transthoracic echocardiogram. Almost all patients had normal left ventricular systolic function, and 23 underwent a previous PV isolation procedure.

**Table 1:** Characteristics of the participants.

Characteristics	Mean ± SD, *n* (%)
Anthropometric parameters	
Age (years old)	68.7 ± 9.5
Men (*n* [%])	66 (64.7)
Height (cm)	163.2 ± 10.0
Weight (kg)	64.6 ± 12.5
CHADS2 score	1.3
Chronic heart disease (%)	25 (24.5)
Hypertension (%)	69 (67.6)
Age > 75 years old (%)	23 (22.5)
Diabetes mellitus (%)	15 (14.7)
Stroke (%)	3 (2.9)
1st session (%)	85 (83.3)
pAF	43 (42.2)
persAF	30 (29.4)
LLAF	4 (3.9)
AFL/AT/PSVT	25 (24.5)
TTE	
LAD (mm)	39.5 ± 7.3
EF (%)	51.7 ± 9.5
ECG	
Sinus rhythm (%)	59 (57.8)

*Note:* Table 1 shows characteristics of the participants at examination of all hypertension was a blood pressure of ≥ 140 mmHg systolic or ≥ 90 mmHg diastolic or use of antihypertensive drugs. Diabetes mellitus was a fasting plasma glucose level of ≥ 7.0 mmol/L or use of antidiabetic agents.

### 3.2 Left Atrial Anatomy

Although there was some slight deterioration in image quality in the 3D images reconstructed from the early and delayed phases, there was no case of deterioration in image quality to the extent that it would make evaluation difficult. Graphical Abstract shows the representative 3D images of the LA and PV. In the early phase, 85 (83.3%) patients exhibited a typical PV pattern with separate ostia for the upper and lower PVs. The left common trunk (LCT) was observed in 12 (11.8%) patients, the right common trunk (RCT) was observed in one patient, and four patients had a right middle PV (RMPV). No patients underwent LCT, RMPV, or RCT simultaneously. We observed the early branching of the right superior PV (RSPV) in eight (7.8%) patients and right inferior PV (RIPV) in nine (8.8%) patients. Six of them had simultaneous early branching of the RSPV and RIPV. One patient experienced early branching of the left superior PV (LSPV). We found accessory appendages in two cases. No differences were observed in the determination of the anatomical variations during the delayed phase. The kappa statistic for evaluating the degree of agreement in detecting PV variations in the early and delayed phases was 1.0 because the variations were consistent. The demographic left atrial anatomy is summarized in Table 2.

**Table 2:** Anatomical variations.

Anatomical variations	CCT phase	
	Early	Delayed
Typical PV pattern	85	85
LCT	12	12
RCT	1	1
RMPV	4	4
Early branching		
LSPV	1	1
LIPV	0	0
RSPV	8	8
RIPV	9	9
Roof vein	5	5
Accessary appendage	2	2

*Note:* Table 2 shows the anatomical variations between early phase and delayed phase.

### 3.3 PV Diameter and Inferior PV Position Relative to the Anterosuperior Edge of Th8

**Figure 2:**
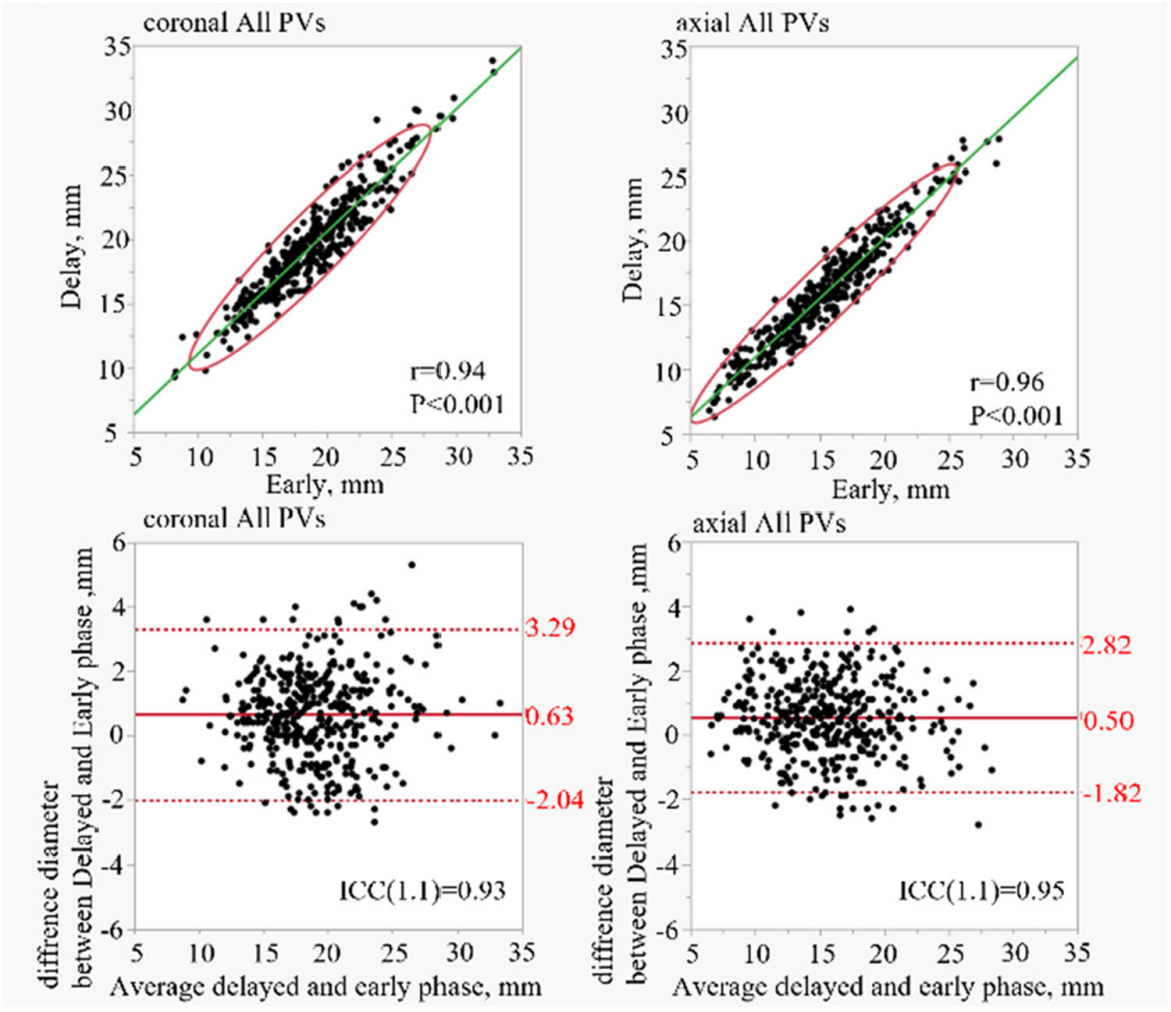
Shows the correlation coefficient and Bland−Altmann plot of all axial and coronal PVs diameter. The Bland−Altmann plot shows the difference in diameter measurements between the delayed and early phases of all PVs. ICC refers to Intra‐class coefficient correlation. The upper and lower red bars show the 95% agreement limits as ±1.96 SD.

**Figure 3:**
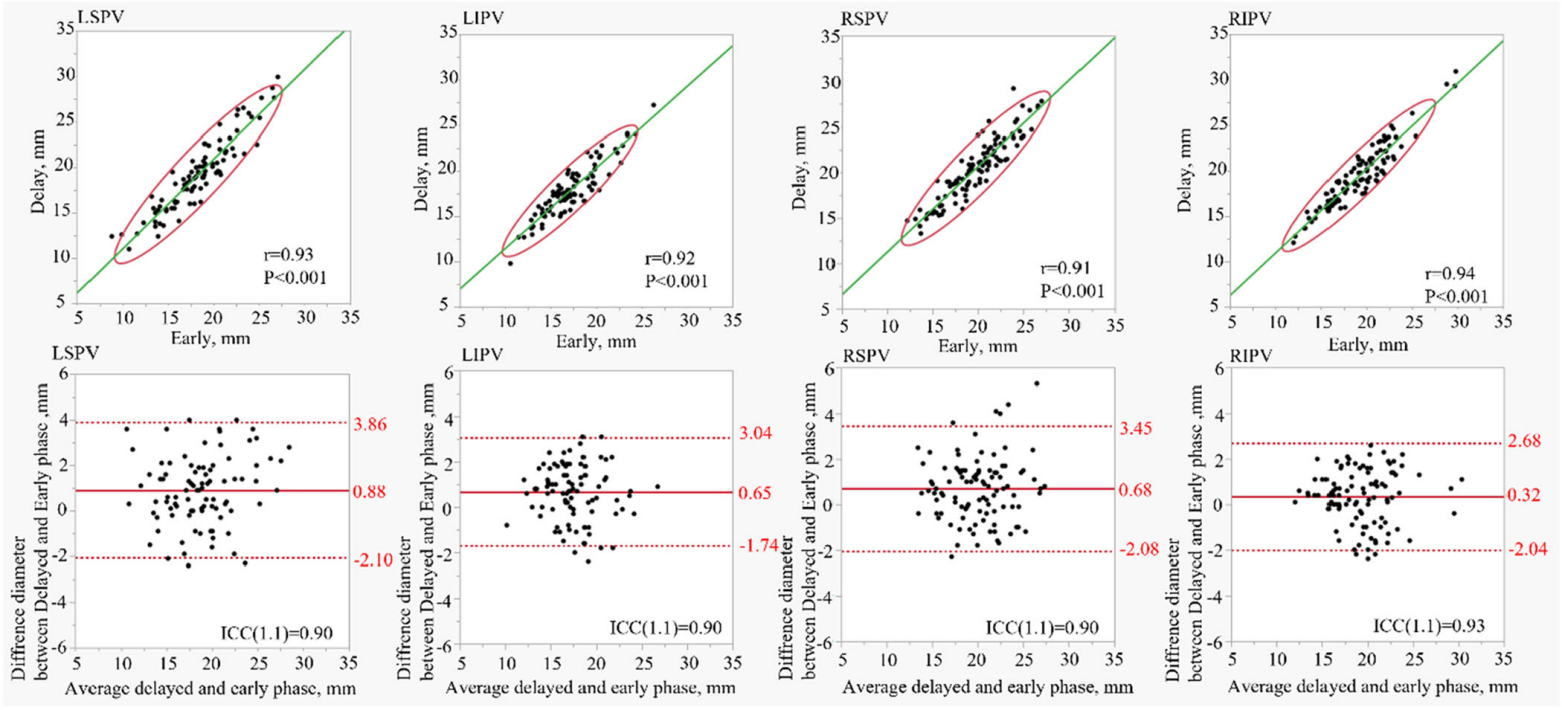
Shows the correlation coefficient and Bland−Altmann plot of each coronal PVs diameter. The Bland−Altman plot shows the difference in diameter measurements between the delayed and early phases of all PVs. ICC refers to Intra‐class coefficient correlation. The upper and lower red bars show the 95% agreement limits as ±1.96 SD.

**Figure 4:**
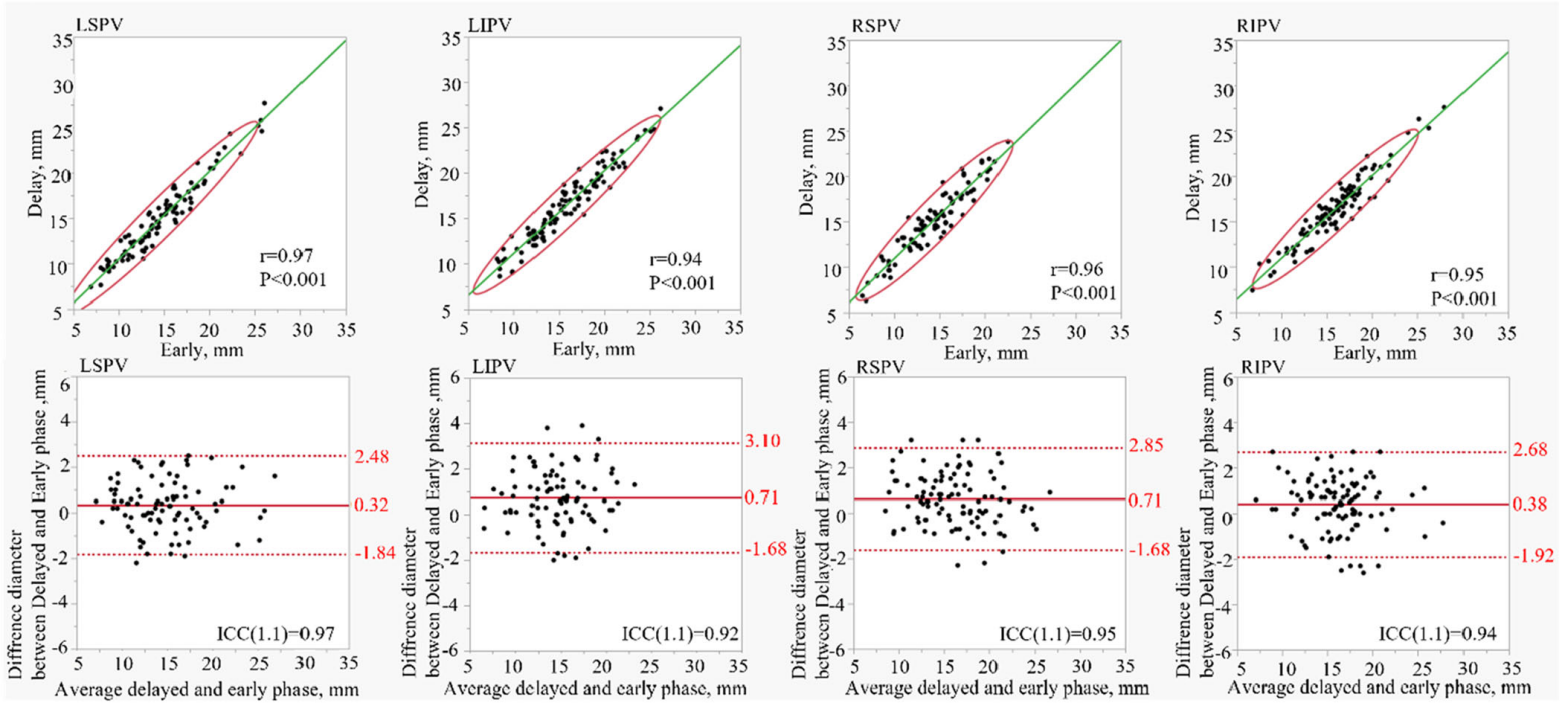
Shows the correlation coefficient and Bland−Altmann plot of each axial PVs diameter. The Bland−Altmann plot shows the difference in diameter measurements between the delayed and early phases of all PVs. ICC refers to Intra‐class coefficient correlation. The upper and lower red bars show the 95% agreement limits as ±1.96 SD.

The early and delayed phases were measured independently, resulting in 399 PVs. In the coronal plane, all PV diameter was 18.71 ± 3.83 mm in the early phase and 19.34 ± 3.88 mm in the delayed phase. The axial images show a diameter of 15.35 ± 4.21 mm in the early phase and 15.84 ± 4.08 mm in the delayed phase. The mean differences between the early and delayed phases were calculated, with values of 0.63 ± 1.36 mm and 0.50 ± 1.2 mm for the coronal and axial planes, respectively. The correlation coefficients were *r* = 0.94 and *r* = 0.96 for the coronal and axial planes, respectively. Figure 2 shows the correlation coefficients for each PV and the Bland–Altman analysis results. Furthermore, individual PVs were analyzed. The correlation coefficients in the coronal view for the LSPV, left inferior PV (LIPV), RSPV, and RIPV were 0.93, 0.92, 0.91, and 0.94, respectively. The mean differences for these veins were 0.88 ± 1.52 mm (LSPV), 0.65 ± 1.22 mm (LIPV), 0.68 ± 1.4 mm (RSPV), and 0.32 ± 1.2 mm (RIPV). Similarly, the correlation coefficients for the axial views of the LSPV, LIPV, RSPV, and RIPV were 0.97, 0.94, 0.96, and 0.95, respectively. The corresponding mean differences were 0.32 ± 1.10 mm (LSPV), 0.71 ± 1.22 mm (LIPV), 0.60 ± 1.1 mm (RSPV), and 0.38 ± 1.2 mm (RIPV). The volume of interest and cross‐sectional area analyses revealed no significant differences between the early and delayed phases. Figure 3 provides the correlation coefficients and the results of the Bland−Altman analysis of each PV for the coronal view, and Figure 4 provides the axial view. In all analyses, an ICC of over 0.90 was found.

We measured the distance between the anterosuperior edge of the Th8 and the bottom of the inferior PVs, using Th8 as a reference point. The position of the bottom of the inferior PVs was assessed along three axes: coronal, sagittal, and vertical. For the LIPV, the measurement errors were 1.44 ± 0.99 mm (coronal), 1.20 ± 1.04 mm (sagittal), and 1.17 ± 1.69 mm (vertical). For the RIPV, the errors were 1.28 ± 0.95 mm (coronal), 0.95 ± 0.70 mm (sagittal), and 1.27 ± 1.81 mm (vertical). The detailed results are presented in Table 3.

**Table 3:** PVs diameter and the position of the bottom of inferior PVs.

A All PVs							
		Coronal, mm		Axial, mm		VOI	CSA, mm^2^
*n* = 399	Early	18.71 ± 3.83		15.35 ± 4.21		0.78 ± 0.16	231.0 ± 98.3
	Delayed	19.34 ± 3.88		15.84 ± 4.08		0.79 ± 0.15	246.4 ± 102.4
	Δ	0.63 ± 1.36	*p* < 0.0001	0.50 ± 1.2	*p* < 0.0001		

B LPV							
		Coronal, mm		Axial, mm		VOI	CSA, mm^2^
LSPV *n* = 90	Early	18.33 ± 3.77		14.74 ± 4.34		0.77 ± 0.18	216.17 ± 94.91
	Delayed	19.21 ± 4.01		15.05 ± 4.34		0.76 ± 0.18	231.56 ± 103.40
	Δ	0.88 ± 1.52	*p* < 0.0001	0.32 ± 1.10	*p* < 0.0001		
LIPV *n* = 90	Early	17.12 ± 3.03		14.41 ± 3.52		0.81 ± 0.16	196.73 ± 71.61
	Delayed	17.77 ± 2.95		15.12 ± 3.61		0.81 ± 0.15	214.05 ± 74.81
	Δ	0.65 ± 1.22	*p* < 0.0001	0.71 ± 1.22	*p* < 0.0001		
LCT *n* = 12	Early	24.58 ± 5.84		20.38 ± 6.21		0.78 ± 0.14	407.19 ± 160.89
	Delayed	25.11 ± 6.11		20.62 ± 5.12		0.84 ± 0.15	421.87 ± 166.66

C RPV							
		Coronal, mm		Axial, mm		VOI	CSA, mm^2^
RSPV *n* = 101	Early	20.44 ± 3.45		15.87 ± 4.21		0.77 ± 0.17	249.4 ± 91.1
	Delayed	19.75 ± 3.35		16.48 ± 4.02		0.78 ± 0.16	268.1 ± 95.4
	Δ	0.68 ± 1.41	*p* < 0.0001	0.60 ± 1.15	*p* < 0.0001		
RIPV *n* = 101	Early	19.15 ± 3.42		15.98 ± 3.74		0.79 ± 0.13	244.6 ± 90.0
	Delayed	19.47 ± 3.41		16.36 ± 3.59		0.81 ± 0.12	254.9 ± 92.7
	Δ	0.32 ± 1.20	*p* < 0.0001	0.38 ± 1.17	*p* < 0.0001		
RCT *n* = 1	Early	11.8		18.5		0.64	171.4
	Delayed	12.5		18.3		0.68	179.6
RMPV *n* = 4	Early	11.45 ± 2.49		9.55 ± 1.97		0.84 ± 0.1	81.7 ± 32.8
	Delayed	12.53 ± 2.41		10.33 ± 1.88		0.83 ± 0.1	95.0 ± 32.0

D The position of the bottom of the inferior PVs from anterosuperior Th8.							
		Coronal, mm		Sagital, mm		Vertical, mm	
LIPV *n* = 85	Early	31.35 ± 9.59		11.16 ± 7.49		7.94 ± 5.43	
	Delayed	31.44 ± 9.52		11.34 ± 7.40		7.70 ± 5.78	
	Δ	1.44 ± 0.99	*p* < 0.0001	1.20 ± 1.04	*p* < 0.0001	1.17 ± 1.69	*p* < 0.0001
RIPV *n* = 85	Early	15.71 ± 6.44		8.29 ± 4.67		8.01 ± 5.39	
	Delayed	16.02 ± 6.31		8.08 ± 4.52		7.71 ± 5.21	
	Δ	1.28 ± 0.95	*p* < 0.0001	0.95 ± 0.70	*p* < 0.0001	1.27 ± 1.81	*p* < 0.0001

*Note:* Table 3 presents the PV diameters in the coronal and axial views, as well as the position of the bottom of the inferior PVs. These positions were measured in three anatomical axes using Th8 as a reference point. VOI and CSA were calculated based on the PV diameters. *p* values are for paired differences of PV ostial diameters by subtracting delayed phase from early phase.

Abbreviations: CSA, cross‐sectional area; VOI, venous ostium index.

### 3.4 Radiation Dose and CT Value

**Figure 5:**
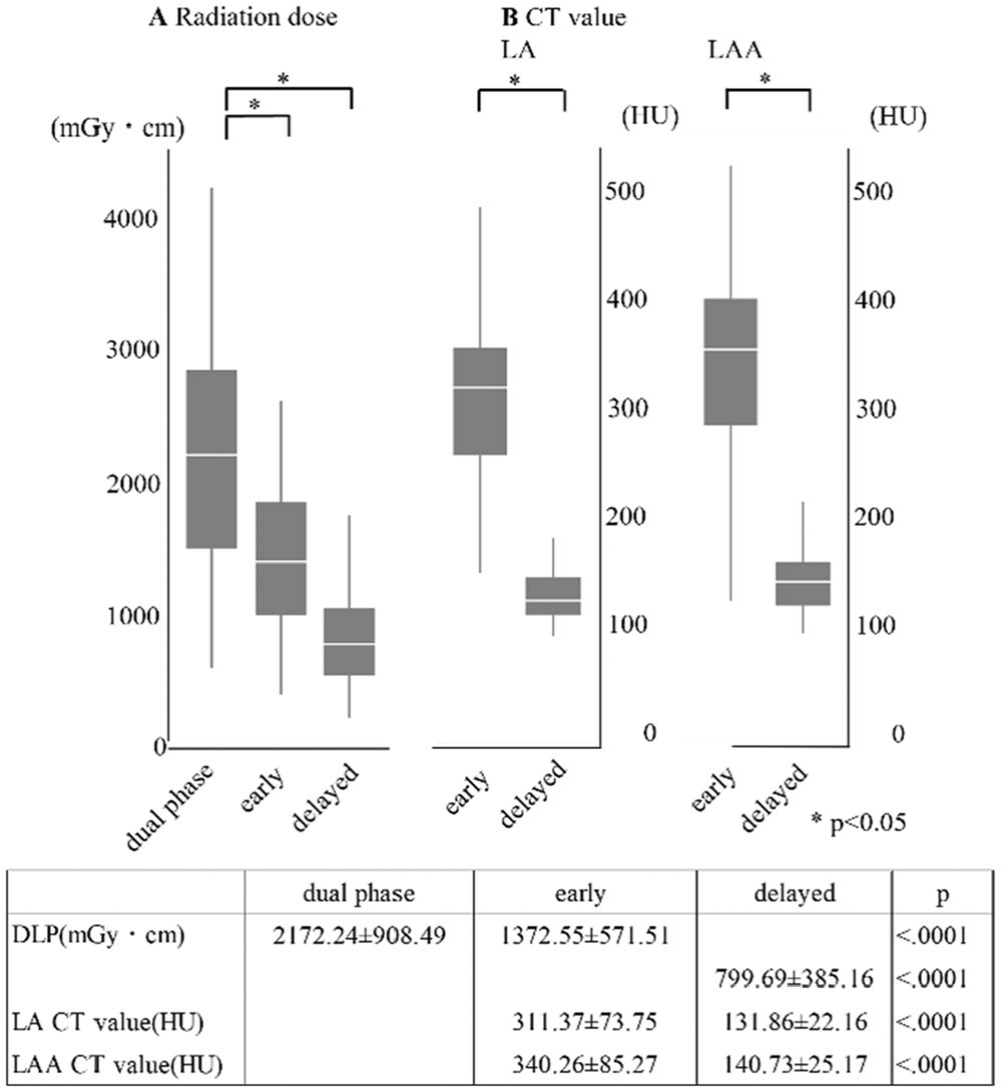
Shows the radiation dose (A) and CT value (B) between dual, early, and delayed phases in the box plot. DLP, dose length product; LA, left atrium; LAA, left appendage.

Analysis was performed for the total exposure dose in the early and delayed phases. The total exposure dose was 2172.24 ± 908.49 mGy‐cm in the dual phase, 1372.55 ± 571.51 mGy‐cm in the single early phase, and 799.69 ± 385.16 mGy‐cm in the single delayed phase. Compared with dual‐phase imaging, single‐phase imaging resulted in significantly lower doses (*p* < 0.05). CT values for the LA were 311.37 ± 73.75 HU in the early phase and 131.86 ± 22.16 HU in the delayed phase, and for LAA, 340.26 ± 85.27 HU in the early phase and 140.73 ± 25.17 HU in the delayed phase. Figure 5 presents the radiation dose data for both phases.

### 3.5 Cardiac Rhythm During CCT Imaging

Analysis was also performed to compare whether the cardiac rhythm at the time of measurement was sinus rhythm or AF. The results are presented in Tables 4 and 5. The AF group tended to have a higher CHADS2 score and larger left atrial diameter, but the difference was insignificant. No significant differences were observed in radiation or absorbed doses (Table 4). When the analysis was performed for each PV, the delayed‐phase diameter tended to be larger in all PVs. However, no significant differences were observed between the groups (Table 5). Additionally, no significant differences were observed in the position of the bottom of the inferior PVs relative to the anterosuperior edge of Th8 based on cardiac rhythm (Table 5).

**Table 4:** Difference in background due to the rythme.

A Background			
	SR	AF	*p*
Male (%)	67.4	62.7	0.76
Age (years)	67.41 ± 9.87	70.37 ± 8.72	0.36
CHADS2 score	1.08 ± 0.94	1.67 ± 0.94	0.50
LVEF (%)	54.50 ± 6.39	47.74 ± 11.55	0.53
LA diameter (mm)	37.63 ± 5.99	42.05 ± 8.08	0.57

B Radiation dose and CT value				
		SR	AF	*p*
DLP (mGy·cm)	Early	1365.20 ± 588.95	1382.63 ± 553.43	0.92
	Delayed	786.83 ± 391.53	834.63 ± 398.97	0.31
LA CT value (HU)	Early	304.63 ± 72.28	320.61 ± 75.58	0.20
	Delayed	133.00 ± 23.05	130.30 ± 21.04	0.48
LAA CT value (HU)	Early	330.00 ± 88.18	354.08 ± 80.13	0.88
	Delayed	140.86 ± 24.44	140.39 ± 26.60	0.33

*Note:* Table 4 shows the difference in the background, radiation dose, and CT value between SR and AF during CCT imaging.

Abbreviations: AF, atrial fibrillation; DLP, dose length product; LA, left atrium; LAA, left appendage; SR, sinus rhythm.

**Table 5:** Difference in PV diameter and the position of bottom of the inferior PVs due to the rhythm.

A All PVs							
		Coronal, mm			Axial, mm		
		SR	AF		SR	AF	
All PVs	Early	18.34 ± 3.62	19.23 ± 4.08		14.99 ± 3.93	15.84 ± 4.55	
	Delayed	19.00 ± 3.72	19.81 ± 4.05		15.41 ± 3.81	16.46 ± 4.39	
	Δ	0.42 ± 1.24	0.42 ± 1.25	*p* = 0.44	0.42 ± 1.24	0.61 ± 1.09	*p* = 0.44

B LPV							
		Coronal, mm			Axial, mm		
		SR	AF		SR	AF	
LSPV	Early	18.25 ± 3.74	18.47 ± 3.85		19.27 ± 4.10	19.12 ± 3.90	
	Delayed	14.64 ± 3.88	14.89 ± 5.07		14.84 ± 3.92	15.40 ± 5.00	
	Δ	1.02 ± 1.50	0.65 ± 1.55	*p* = 0.91	0.20 ± 1.12	0.51 ± 1.06	*p* = 0.75
LIPV	Early	16.74 ± 2.90	17.74 ± 3.18		14.52 ± 3.15	14.23 ± 4.11	
	Delayed	17.46 ± 2.94	18.29 ± 2.92		15.04 ± 3.19	15.26 ± 4.25	
	Δ	0.71 ± 1.17	0.55 ± 1.31	*p* = 0.94	0.52 ± 1.27	1.03 ± 1.07	*p* = 0.41

C RPV							
		Coronal, mm			Axial, mm		
		SR	AF		SR	AF	
RSPV	Early	19.92 ± 3.45	19.52 ± 3.23		15.57 ± 4.09	16.30 ± 4.38	
	Delayed	20.61 ± 3.45	20.20 ± 3.48		0.69 ± 1.39	17.00 ± 4.17	
	Δ	0.53 ± 1.14	0.70 ± 1.15	*p* = 0.43	0.53 ± 1.14	0.70 ± 1.15	*p* = 0.43
RIPV	Early	18.67 ± 3.25	19.82 ± 3.59		15.30 ± 3.89	16.93 ± 3.34	
	Delayed	18.86 ± 3.17	20.32 ± 3.58		15.67 ± 3.80	17.32 ± 3.07	
	Δ	0.19 ± 1.11	0.50 ± 1.31	*p* = 0.35	0.37 ± 1.30	0.39 ± 0.97	*p* = 0.41

D The position of the bottom of the inferior PVs from anterosuperior Th8.										
		Coronal, mm			Sagital, mm			Vertical, mm		
		SR	AF		SR	AF		SR	AF	
LIPV	Early	32.05 ± 10.45	30.35 ± 8.48		10.71 ± 7.23	11.80 ± 7.99		8.50 ± 5.68	7.17 ± 5.14	
	Delayed	32.11 ± 10.53	30.53 ± 8.20		11.08 ± 7.19	11.68 ± 7.87		8.38 ± 5.93	6.74 ± 5.58	
	Δ	1.61 ± 0.81	1.86 ± 0.87	*p* = 0.89	1.51 ± 1.09	1.31 ± 0.82	*p* = 0.56	1.25 ± 1.73	1.61 ± 1.81	*p* = 0.61
RIPV	Early	15.00 ± 6.33	16.36 ± 6.44		7.86 ± 4.08	8.94 ± 5.39		7.25 ± 4.54	9.25 ± 6.14	
	Delayed	14.93 ± 5.89	17.16 ± 6.49		7.69 ± 4.16	8.65 ± 5.03		7.31 ± 4.59	8.44 ± 5.87	
	Δ	1.48 ± 0.65	1.59 ± 0.95	*p* = 0.56	1.09 ± 0.64	1.18 ± 0.57	*p* = 0.15	1.06 ± 1.58	2.14 ± 2.10	*p* = 0.19

*Note:* Table 5 shows the difference in PV diameter and the position of bottom of the inferior PVs between SR and AF during CCT imaging. The *p* value represents the difference in ostial diameter and the distance of the bottom of inferior PVs, which is calculated by subtracting the early phase from the delayed phase.

Abbreviations: AF, atrial fibrillation; SR, sinus rhythm.

### 3.6 LAA Thrombosis

No complications of cerebral embolism occurred after ablation in any patient. In the single‐early‐phase, seven of the 102 patients did not have adequate flow into the LAA, making it difficult to exclude the presence of a thrombus. In the single‐delayed‐phase, inadequate contrast opacification of the LAA was observed in four of the 102 patients—all of whom were among the seven initially suspected of having thrombus. Subsequent TEE confirmed true LAA thrombi in three of these four patients (Central Illustration 1).

**Central Illustration 1:**
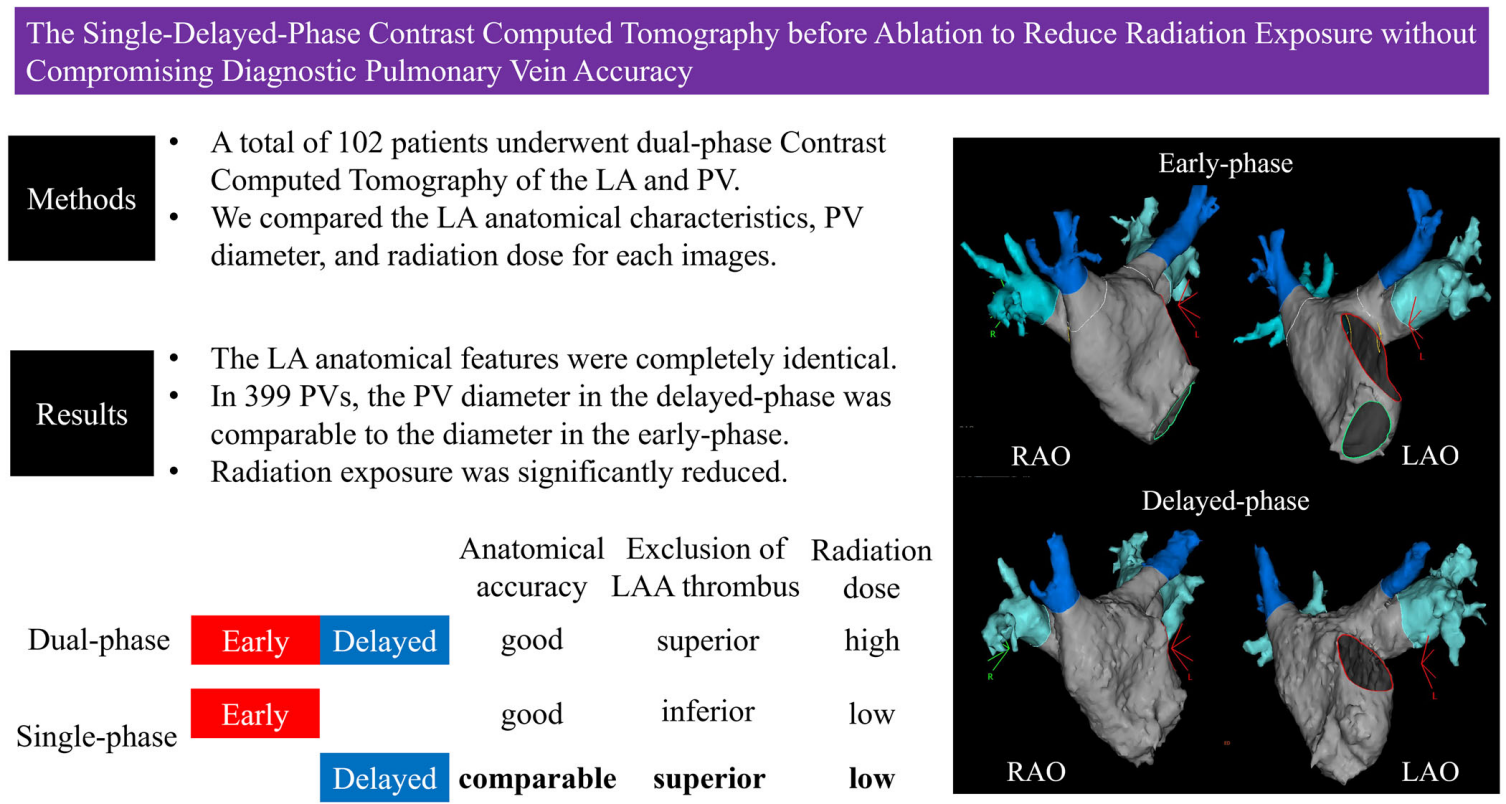
The results of analyzing the early and delayed phases of contrast computed tomography (CCT) images showed that the single‐delayed‐phase was comparable in evaluating the anatomy and diameter of the pulmonary veins to the single‐early‐phase. The single‐delayed‐phase CCT was considered to be effective for evaluation before ablation.

## 4 Discussion

Dual‐phase CCT is an imaging technique commonly used to evaluate vascular structures of the liver and cerebral vessels. Under certain conditions, delayed‐phase CCT provides better diagnostic information than early‐phase CCT in certain organs [10]. However, no study has compared the early and delayed phases of cardiac anatomy assessment. This is the first observational study to compare delayed‐phase CCT with early‐phase CCT in assessing anatomical changes and PV diameters. This study aimed to evaluate the potential of moving from dual‐phase to single‐delayed‐phase scanning to streamline the CCT protocol for the ablation of AF and to determine whether this reduction would adversely affect diagnostic accuracy and treatment planning. The results showed that delayed‐phase CCT accurately identified all anatomical changes and that the PV diameter measurements were comparable to those obtained from early‐phase CCT. The outline is shown in the Graphical Abstract.

The role of CCT in preprocedural planning is still debated, especially considering the potential radiation risks associated with its use [11]. However, with promising applications, such as the evaluation of pericardial fat, CCT is expected to continue to be important in the future [12]. Understanding the morphology of the PVs is important for optimizing ablation strategies. Additionally, cryoballoon and pulsed‐field ablation devices, which have been adopted recently, are simple and effective in reducing the procedure time. However, the shape of the device is expected to limit this technique depending on the morphology and size of the PV [13]. Furthermore, it is important to observe the shape of the PV before the procedure objectively. The PVs can become narrowed after ablation, and mild narrowing is estimated to occur in approximately 20% of patients [14]. Because obtaining images before the procedure allows for the objective evaluation of narrowing after the procedure, the importance of preoperative imaging diagnosis is still considered high. Our results showed that a simplified CCT protocol with single‐delayed‐phase scan did not compromise the accuracy of assessing the morphology and size of the PVs. Furthermore, this approach reduces radiation exposure and streamlines the assessment process. Within the range of error in PV diameter shown in this study, it is unlikely that it will affect the choice of device used in treatment, and it is thought that sufficient information on variations that should be noted in the procedure can be obtained. There is no change in the other procedures when using images created from the delayed phase during actual ablation procedures. In this study, image construction was performed in all cases using CARTO3, and there was no effect on anatomical evaluation, but the image quality of the 3D composite image deteriorated. However, this deterioration in image quality is thought to be compensated for by the electrophysiological mapping performed before the procedure, and it is expected that this will not be a significant problem in actual clinical practice. Still, additional prospective studies will be needed to prove this. Conversely, some centers utilize anatomical information other than PV, such as left atrial wall thickness, roof vein, accessory appendage, and coronary arteries, to consider the ablation strategy. In the present study, we were able to confirm the presence of accessory appendages and roof veins equally in the delayed phase but were not able to examine whether detailed information such as coronary arteries and left atrial wall thickness could be obtained in the delayed phase as in the early phase. Therefore, the use of single delayed phase is recommended for preoperative evaluation of especially simple PVI, such as PFA and balloon ablation. However, it should be noted that it is not clear whether the information from single delayed phase is sufficient when information on coronary artery and left atrial wall thickness is required.

The potential value of single‐delayed‐phase CCT for identifying LAA thrombi before AF ablation is clear. Although low, the prevalence of LAA thrombosis in anticoagulated patients before AF ablation is significant enough to warrant comprehensive preprocedural screening [15]. TEE remains the gold standard for detecting LAA thrombi; however, its invasiveness and associated risk of complications have prompted a reevaluation of imaging strategies [16, 17]. The risks associated with TEE, including esophageal injury and gastrointestinal bleeding, emphasize the need for the development of safer but equally effective alternatives. A meta‐analysis conducted by Romero et al. compared the diagnostic accuracies of early‐phase CCT, delayed‐phase CCT, and TEE for LAA thrombus detection, focusing on the sensitivity of these methods in a clinical setting [18]. The results demonstrated that single‐early‐phase CCT was inadequate to exclude LAA thrombus. In contrast, delayed‐phase CCT exhibits 100% thrombus detection sensitivity. In this study, LAA thrombi were evaluated in both the early and delayed phases, with at least 1 day between evaluations to prevent bias—consistent with the approach used for anatomical evaluation. In general, comparing early‐ and delayed‐phase images can help differentiate between LAA thrombus and slow blood flow. However, a recent study directly compared the sensitivity and specificity of thrombus detection between single‐early and single‐delayed‐phase CCT, reporting 100% sensitivity in the single‐delayed [19]. Therefore, we consider that omitting the early phase would not compromise the diagnostic utility of LAA thrombus exclusion. These findings suggest that single‐delayed‐phase CCT is a viable alternative to more invasive procedures without compromising diagnostic accuracy. Conversely, a single‐early‐ phase CT scan is insufficient to achieve this purpose. Our study demonstrated that delayed‐phase CCT can be employed to evaluate PVs before ablation and can be considered a potential routine approach for obtaining information crucial for ablation.

The findings of this study indicate a tendency toward increased PV diameter during the delayed phase. This is because the diameter of the left atrial side may have been measured at the time of measurement. In the delayed phase, the lumen and vessel wall boundaries were unclear compared with those in the early phase. Consequently, the measured value may be obtained outside the actual boundary. Furthermore, even when the measurement is conducted per the defined protocol, errors are highly probable if the LA and PV are connected smoothly. This error was not contingent on the specific phase under examination, suggesting that the effect of the indistinct boundary on the vessel wall was substantial. However, the difference was not significant. The influence of these differences on treatment outcomes requires further investigation. The accuracy of 3D merging during CT and ablation procedures has been demonstrated to be approximately ±2 mm [20]. Additionally, the positional relationship of the bottom of the inferior PVs is considered essential by the operator. To evaluate this with high accuracy, we used the anterosuperior edge of Th8 as a reference point, as it is unaffected by contrast phase and can be consistently identified. Consequently, the maximum measurement error was only 1.44 mm, which we considered the affection as minimal. Therefore, it can be concluded that the degree of difference in this study has little clinical impact. This accuracy was demonstrated to be independent of the cardiac rhythm during CCT imaging. It is anticipated that patients with AF will experience more difficulty in providing accurate motivation when undergoing electrocardiogram‐synchronized imaging than patients with sinus rhythm. We hypothesized that image quality would be inferior and measurement errors would be more significant in patients with AF. However, the results were not significantly different from those for sinus rhythm. This may be attributed to inadequate contraction of the LA and the PV during AF, which is a vascular vessel. Nevertheless, sufficient anatomical information could be obtained with delayed‐phase CCT, irrespective of the rhythm. Parameters such as left atrial volume and wall thickness are valuable for guiding ablation beyond PV isolation, but further research is needed to evaluate these measurements specifically in the delayed phase. Notably, this study was retrospective, and no ablation procedures were performed using the 3D model. However, other studies have assessed the time required for segmentation, procedure duration, and fluoroscopy time when using 3D models generated from simple CT compared to contrast‐enhanced CT [21, 22]. These studies found that although segmentation time is slightly longer with plane CT, the overall procedure and fluoroscopy times are not inferior to those using contrast CT. Based on these findings, we believe that procedures can be performed using 3D images obtained from delayed‐phase CT in a manner similar to those obtained from the early phase.

The efficacy of these imaging protocols for delayed‐phase CCT requires further investigation. A consensus has yet to be reached regarding the optimal imaging protocol for delayed‐phase CCT [6, 23]. In this study, delayed‐phase imaging was uniformly performed 1 min after contrast injection to standardize conditions. In contrast, a recent study [19] suggests a direct 3 min delayed scan for improved detection of LAA thrombus in high‐risk patients. However, this longer delay is expected to significantly reduce image quality for catheter ablation planning compared to the 1 min delay. Further research is warranted to determine the optimal for delayed‐phase imaging. At our institution, the early phase can be readily omitted because it is defined as 1 min after contrast injection and is not contingent on the early phase. If pre‐ablative assessment is conducted using single‐delayed‐phase imaging, the imaging range will be identical to that of the early phase of this protocol. Consequently, a slight increase in the delayed‐phase dose values was expected, whereas the reduction in the radiation dose in single phase remained unchanged. Conversely, if the single‐delayed‐phase is used, there may be compatibility issues between the results obtained using images to evaluate the coronary arteries and those obtained in the early phase. Previous studies have indicated that coronary artery lesions can be effectively assessed using preoperative early‐phase CCT [24]. However, the prevalence of these lesions is relatively low, and the necessity for routine assessment remains debatable. This study identified several predictive risk factors of myocardial ischemia. In patients with these risk factors, routine imaging procedures may be preferable. In the latter case, CCT was the most commonly used method for assessing PV stenosis. When evaluating PVs that have historically been evaluated in the early phase, if it is possible to measure PV diameter equally in the early and delayed phases, it may also reinforce the fact that there are almost no disadvantages in the delayed phase.

### 4.1 Limitation

Our study has some limitations. First, only a small number of patients were included. Second, there are potential changes in PV diameter based on position and angle, along with the inability in directly comparing the accuracy with electroanatomical mapping. It is difficult to eliminate the effects of heart rate and contrast completely; therefore, the locations deemed appropriate in each phase of the heart cycle and the imaging angles may not be entirely consistent. Because this was a retrospective study, delayed‐phase 3D models were not used in actual clinical practice, and it was impossible to investigate the problems that might occur if they were used in actual treatment. Therefore, future randomized prospective studies are warranted. Finally, the current study only included data from a single center. Replications with other studies are required to confirm our findings.

## 5 Conclusion

The anatomical information obtained from delayed‐phase CCT was not inferior to that obtained from early‐phase CCT. Single‐delayed‐phase CCT can provide sufficient anatomical information before ablation. Given the diagnostic accuracy and low‐risk profile of delayed‐phase CCT, it is a promising method for reducing early‐phase CT scans for both anatomical assessment and LAA thrombus detection in candidates for AF ablation.

## Author Contributions

Dr. Yasushi Oginosawa reviewed and approved of this study. Dr. Masaharu Kataoka, the departmental head, helped to develop it. Other authors helped with data collection and calculations.

## Ethics Statement

This study was approved by the University of Occupational and Environmental Health Ethics Review Board. All procedures were performed in accordance with the principles of the Declaration of Helsinki of 1964 performed all procedures.

## Consent

As this was a retrospective study, the Institutional Review Board waived the requirement for written informed consent.

## Conflicts of Interest

The authors declare no conflicts of interest.

## Data Availability

The deidentified participant data from this study will not be shared for several reasons, including data protection and the risk of re‐identification.
